# Health and Quality of Life of Individuals with Dental Conditions during COVID-19 Lockdown in Jordan

**DOI:** 10.3290/j.ohpd.b3601673

**Published:** 2022-11-23

**Authors:** Zeid A. Al-Hourani, Khader A. Almhdawi, Isra A. AlBakri, Anas N. Alibrahim, Donia Obeidat

**Affiliations:** a Associate Professor of Dental Technology, Department of Applied Dental Sciences, Faculty of Applied Medical Sciences, Jordan University of Science and Technology, Irbid, Jordan. Principle investigator, research idea, research methods, wrote the manuscript.; b Professor of Occupational Therapy and Rehabilitation Sciences, Department of Rehabilitation Sciences, Faculty of Applied Medical Sciences, Jordan University of Science and Technology, Irbid, Jordan. Research methods, evaluated the data, revised the manuscript.; c Assistant Professor of Dental Technology, Department of Applied Dental Sciences, Faculty of Applied Medical Sciences, Jordan University of Science and Technology, Irbid, Jordan. Collected the data, revised the manuscript.; d Assistant Professor of Prosthodontics, Department of Prosthodontics, Faculty of Dentistry, Jordan University of Science and Technology, Irbid, Jordan. Collected the data, revised the manuscript.; e Research and Teaching Assistant in Rehabilitation Sciences, Faculty of Applied Medical Science, Jordan University of Science and Technology, Irbid, Jordan. Performed statistical analysis.

**Keywords:** corona virus, dental health care, pandemic, quarantine

## Abstract

**Purpose:**

To assess the level of health-related quality of life (HRQoL) and its predictors among individuals with dental conditions requiring treatment during the COVID-19 pandemic in Jordan, and to highlight COVID-19 impacts on these individuals’ mental health and dental care.

**Materials and Methods:**

A cross-sectional online-based survey was conducted. The study questionnaire was composed of items related to stress during COVID-19. Descriptive analyses were used to describe the study’s main outcome measures, and multiple variable regression analysis was conducted to identify the quality of life predictors.

**Results:**

Participants’ HRQoL mean scores as measured by SF-12 were 69.5 (± 19.3) for the physical component and 58.9 (± 21.1) for the mental component. Mean scores for depression, anxiety, and stress measured by DASS21 were 10.1, 7.8, and 11.7, respectively. The regression model showed depression (β = -0.76 [95%CI -0.92 to -0.59], p < 0.001), stress (β = -1.74 [95%CI -2.53 to -0.94], p < 0.001), and oral HRQoL (β = -1.25 [95%CI -1.95 to -0.55], p = 0.001) as statistically significant negative predictors for HRQoL. Finally, family income was identified as positive significant predictor for HRQoL (β = 2.51 [95%CI 0.06 to 4.95], p = 0.045).

**Conclusion:**

This study found that patients with dental issues had a low quality of life and a high level of mental health symptoms during COVID-19 in Jordan. Healthcare policymakers should consider the continuation of dental services when planning for similar emergencies in the future, particularly if accompanied by lockdowns.

In keeping with the World Health Organization’s (WHO) definition of health, the integrity of oral health is considered an essential element^39^ and primary factor influencing an individual’s well-being, as oral health has a high impact on daily activities such as eating, speaking, and aesthetics.^13^ The socio-environmental model of health suggests that health is the capability for optimal functioning and social and psychological well-being.^40^ Oral health has been defined as 1. the state of the mouth and other associated structures where a current disease is contained and future illness is prevented, 2. the ability to masticate food with appropriate occlusion, and 3. acceptable aesthetics.^23^ According to Locker,^18^ concepts of health and quality of life are difficult to define, multidimensional and complex, predominantly subjective, constantly evolving, and vary according to social, cultural, political and practical contexts. He also stated that general health and oral health are highly interconnected.^5^ In the early 1960s, the concept of Oral Health-Related Quality of Life (OHRQoL) was introduced. It was related to the broader picture of health and well-being after including dental diseases in the classical “sick role” theory.^38^ Sick role theory defines the deviant role of the ill person, which may be against some societal expectation and differs from that of the non-ill person. However, today’s lifestyle-centered health promotion and increased prevalence of chronic diseases delimit the role of sick role theory in the medical system.

The concept of OHRQoL has added more opportunities for dental clinicians and researchers to perform clinical trials and epidemiological studies. Several studies demonstrated that periodontal diseases might have a negative impact on general oral health and indicate low OHRQoL and periodontal status among dental patients.^1,15^ One study^1^ researched the severity of dental and oral conditions and their impact on the quality of life. Those authors highlighted the significance of OHRQoL for the clinical practice of dentistry, dental research and dental-school curricula. The authors stated that OHRQoL should be a fundamental part of developing health programs and that it should be integrated into healthcare practices for better healthcare outcomes. In agreement with the previously mentioned study,^1^ another group of authors stated that OHRQoL might help build dental health care programs by alarming politicians and health policymakers if carried out on a national scale.^29^ It is evident that OHRQoL measures are not alternative to physical examinations, but rather are additional tools that are helpful in comprehensive and client-centered diagnosis and treatment.^18^

Many questionnaires are commonly used for assessing OHRQoL. Previous questionnaires to assess OHRQoL contained multiple items on general and/or specific health measures. Available measures assess the overall oral health, whereas specific criteria assess specific populations with particular conditions, such as edentulous patients, preschool children, people who suffer from caries or malocclusion, etc.^6^ The importance of OHRQoL lies in its connecting oral health with its impact on the individual’s general health.^28^

The Corona virus (COVID-19) pandemic has changed global perspectives on culture, politics, economy and healthcare.^12^ As the number of cases and affected countries escalated, risk assessment of the novel COVID-19 disease was upgraded by the WHO to be of very high importance at a global level.^38^ Developing countries have less than optimal access to dental health care facilities. This access has been reduced even further or suspended in times of curfew during the COVID-19 pandemic. In such an extraordinary era, OHRQoL might be a valuable aspect to measure the impact of oral health on general health and the Quality of Life (QoL).^34^

Measuring OHRQoL may shed light on the association between oral health conditions and QoL, which might raise the awareness of policymakers regarding the impacts of limiting access to oral health care facilities.^1,16^ With the heightened fear of becoming infected in healthcare facilities and limited access to dental facilities during COVID-19, it has become essential to assess patients’ general medical needs and psychological status while addressing the need for dental care.^7^ Throughout the period of the COVID-19 lockdown (March to May, 2020), Jordanian businesses and schools were shut down, and dental care facilities were no exception (except for treating dental emergencies in governmental hospitals).^19,30^

The closure of dental clinics has been attributed to the high risk of infection and transmission of disease, as dental care procedures involving high-speed handpieces and scalers are considered an important source of aerosol transmission of COVID-19 infections.^27^ This study aimed to assess the level of health-related quality of life (HRQoL) and its predictors among individuals with dental conditions requiring treatment during the COVID-19 pandemic in Jordan. Furthermore, COVID-19 impacts on these individuals’ mental health and dental care were highlighted.

## Materials and Methods

### Study Design and Setting

This cross-sectional study was conducted in the cities of Irbid and Amman, Jordan, from June to August, 2020.

### Study Sample and Sampling Techniques

The study used a random sampling technique to recruit participants. According to G-power software,^28^ using an effect size of 0.15, α at 0.05, and assuming 15 predictors, a minimum sample size of 139 participants was required to guarantee a statistical power of 80%. In the wake of restrictions by the Jordanian government to contain the prevalence of COVID-19, an online questionnaire was used via Google Forms. Accounting for possible non-responses, and in order to guarantee the minimum sample required, these forms were e-mailed to 200 individuals who visited the dental clinics of Irbid and Amman. Only the e-mail addresses of the residents of the two cities were picked from the database of dental clinics located in Irbid and Amman. Random function in Microsoft Excel was used to label e-mail address records, then these records were ordered ascendingly and the first 200 were used. This database includes the contact information of hundreds of individuals actively engaged in dental treatment in public and private clinics. Participants were recruited if they were between 18 and 80 years old and had actively visited a dentist for a therapeutic dental need in the last 12 months and still needed follow-ups. Individuals who had undergone major surgeries, had physical or mental diseases diagnosed in the last 12 months, or were in the acute phase of COVID-19 infection were excluded from the study. Out of 200 survey forms sent, 170 were returned, of which 149 were considered eligible. The rest were excluded as they were incomplete; the response rate was thus 74.5%.

### Data Collection

Data were collected through an online questionnaire divided into three parts and developed in Arabic. The first part focused on sociodemographics, e.g. the participants’ background information, including gender, age, residence, education, monthly income. This part also included questions about the main dental complaint, self-assessment of the severity of pain (scale 0-10), whether the participant was diagnosed with any dental or oral condition in the past 10 years and was still under treatment, any other medical issues, if there was a cancellation of an appointment due to the pandemic, type of facility for dental treatment, fear of visiting the dental clinic during the pandemic (scale 0-5), and the effect of dental treatment on the participants’ general health.

Using the OHRQoL-14 questionnaire, the second part focused on participants’ evaluation of the impact of dental and oral cavity pain on daily life over the past month. The reliability of the questionnaire is good, i.e. Cronbach’s α ≥ 0.6, whereas the highest reliability score was found in the “social disability” subscale (α = 0.87).^20^

The third part included the depression, anxiety and stress scale-21 (DASS-21). DASS-21 is a set of three self-report scales designed to measure the emotional states of depression, anxiety and stress. Each of the three DASS-21 scales contains seven items, divided into subscales with similar content. The depression scale assesses dysphoria, hopelessness, devaluation of life, self-deprecation, lack of interest/involvement, anhedonia and inertia. The anxiety scale assesses autonomic arousal, skeletal muscle effects, situational anxiety, and subjective experience of anxious affect. The stress scale is sensitive to levels of chronic non-specific arousal. It assesses difficulty relaxing, nervous arousal and being easily upset/agitated, irritable/over-reactive and impatient. Scores for depression, anxiety and stress are calculated by summing the scores for the relevant items.

The DASS-21 is based on a dimensional rather than a categorical concept of psychological disorder. The internal consistency of the subscales for the 21-item DASS is high, with Cronbach’s alpha of 0.92 (DASS-Anxiety), 0.97 (DASS-Depression), and 0.95 (Dass-Stress).^25^ Similarly, the Construct and Convergent of the subscales has been validated in many studies.^17,32^ The assumption upon which DASS-21 development was based (and was confirmed by research data) is that the differences between depression, anxiety and stress experienced by normal subjects and clinical populations are essentially differences of degree. The DASS-21, therefore, has no direct implications for the allocation of patients to discrete diagnostic categories postulated in classificatory systems such as the DSM and ICD. Recommended cut-off scores for conventional severity labels (normal, mild, moderate, severe, extremely severe) are shown in Table 1. NB scores on the DASS-21 were multiplied by 2 to calculate the final score.

**Table 1:** Cut-off scores for severity categories of depression, anxiety, and stress in DASS-21

	Depression	Anxiety	Stress
Normal	0–9	0–7	0–14
Mild	10–14	8–9	15–18
Moderate	14–20	10–14	19–25
Severe	21–27	15–19	26–33
Extremely severe	+28	+20	+34

The questionnaire was available for only seven days, at the early stage of the pandemic. Responses were obtained and tabulated in Excel (Microsoft; Redmond, WA, USA), then imported to SPSS for statistical analysis. The results were calculated using percentages and frequencies for categorical variables, and means ± SD for continuous variables. Multivariable regression analysis was conducted on factors associated with health-related quality of life, according to the SF-12 survey (by Wade, Kosinksi and Keller 1996; a 12-item questionnaire used to assess generic health outcomes from the patient’s perspective) total score.

### Ethical Consideration

All study procedures were approved by the institutional review board (IRB) at Jordan University of Science and Technology (approval number 127/132/2020). All the participants were briefed about the aim, methodology, risks and usefulness of this research. An informed consent was obtained from all the participants before the commencement of the study. Moreover, the study also assured the participants that their personal information would be kept confidential at all times.

## Results

A total of 149 participants completed the survey, of which 56.4% were females with a mean age of 38.3 (±11.7) years. The majority of the participants (43.6%) were from the city of Irbid; 71.8% of them were employed prior to COVID-19, with the highest percentage reported having an average monthly income between 500 and 1000 Jordanian Dinars (700–1400 $US). Table 2 shows an overview of the general demographics of this study participants.

**Table 2:** Participants’ sociodemographic characteristics

Characteristic		Mean (SD) or N (%)
Age		38.3 (11.7)
Gender	Male	75.84 (50.9%)
	Female	73.15 (49.1%)
Marital status	Single	59.7 (40.1%)
	Married	89.2 (59.9%)
Residence area	Amman	36 (24.2%)
	Irbid	65 (43.6%)
	Other cities	48 (32.2%)
Education	High school or less	12 (8%)
	Community college	25 (16.8%)
	Bachelor’s degree	62 (41.6%)
	Graduate level	50 (33.6%)
Work prior to COVID-19	Yes	107 (71.8%)
	No	42 (28.2%)
Monthly family income (Jordanian dinars)	<250	8.9 (6%)
	250-500	43.4 (29.1%)
	500-1000	51.1 (34.3%)
	>1000	45.6 (30.3%)
Dental care provider	Private practice	125 (83.9%)
	Ministry of Health	7 (4.7%)
	Royal Medical Services	6 (4%)
	University Clinics	11 (7.4%)

The duration of participants’ dental conditions ranged from 1 to 11 years, with caries being the highest (43%) reported diagnosis. Due to COVID-19, 61.7% of participants reported cancelling one or more of their dental appointments. Around two-thirds of the participants reported having a fear of infection due to COVID-19, and 32.9% stated that they would visit the dental clinic only in emergencies in the meantime. Table 3 compares the different responses concerning participants’ dental health and COVID-19.

**Table 3:** Impacts of COVID-19 on participants’ dental health (n = 149)

Duration of disease since diagnosis (years), mean ± SD: 4.29 ± 3.8		
Characteristics		N%
Main dental diagnosis	Missing teeth	14 (9.4%) 11 (7.4%) 64 (43%) 3 (2%) 23 (15.4%) 5 (3.4%) 10 (6.7%) 7 (4.7%) 12 (8.1%) 92 (61.7%) 57 (38.3%) 4 (2.7%) 7 (4.7%) 12 (8.1%) 45 (30.2%) 81 (54.4%) 12 (8.1%) 7 (4.7%) 22 (14.8%) 43 (28.9%) 65 (43.6%) 51 (34.2%) 9 (6.0%) 49 (32.9%) 40 (26.8%)
	Tooth fracture	
	Dental caries	
	Tooth discoloration	
	Gingivitis	
	TMJ pain	
	Prosthetic treatment pain	
	Tooth sensitivity	
	Other	
Dental appointment cancellation due to COVID-19	Yes	
	No	
Dental visit improves my health	Strongly disagree	
	Disagree	
	Neutral	
	Agree	
	Strongly agree	
Fear of infection when visiting dentist during COVID-19	Strongly disagree	
	Disagree	
	Neutral	
	Agree	
	Strongly agree	
Planning on visiting a dentist	Yes	
	No	
	Just in emergencies	
	After controlling COVID-19	

TMJ: temporomandibular joint.

### Health Characteristics of Participants

Based on the cut-off scores listed in Table 1, the participants demonstrated an overall mild depression with a mean score of 10.1 (±9.2), a mild level of anxiety with a mean score of 7.8 (±7.7), and a normal level of stress with a mean score of 11.7 (±9.4) (Table 4). Participants’ HRQoL measured by SF-12 mean scores were 69.5 (± 19.3) for the physical component and 58.9 (± 21.1) for the mental component. Participants’ Oral Health-related Quality of Life mean score was 14.3 (±8.6). Table 4 also shows participants’ mean ± SD toothache score (4.4 ± 3.2) during the previous week on a 10-point visual analogue scale (VAS).

**Table 4:** Health characteristics of participants during COVID-19

Characteristic	Mean ± SD
Depression	10.1 ± 9.2
Anxiety	7.8 ± 7.7
Stress	11.7 ± 9.4
Toothache score (VAS)	4.4 ± 3.2
Oral Health-related Quality of Life	14.3 ± 8.6
Physical component score (SF-12)	69.5 ± 19.3
Mental component score (SF-12)	58.9 ± 21.1
SF-12 total score	63.3 ± 18.8

VAS: Visual Analog Scale for pain rating.

### Factors Associated with Health-Related Quality of Life

The multiple regression model explained 57.3% of the variance in HRQOL (r^2^ = 0.573, F = 43.301, p < 0.001). Stress (β = -0.77 [95%CI -1.23 to -0.31], p = 0.001), depression (β = -0.67 [95%CI -1.08 to -0.26], p = 0.002), and oral health related quality of life (β = -0.42 [95%CI -0.71 to -0.14], p = 0.004) were significant negative predictors of HRQoL. On the other hand, family income (β = 2.51 [95%CI 0.06 to 4.96], p = 0.045) was the only significantly positive predictor of HRQoL. Table 5 demonstrates the regression analysis results.

**Table 5:** Multivariable regression analysis of factors associated with Health-related Quality of Life measured by SF-12 survey, total score

Factor	β coefficient	95% Confidence interval		p-value
Stress	-0.77	-1.23	-0.31	0.001
Depression	-0.67	-1.08	-0.26	0.002
Oral Health-related Quality of Life	-0.42	-0.71	-0.14	0.004
Family income	2.51	0.06	4.96	0.045

## Discussion

To our knowledge, this is the first global study to evaluate HRQoL among dental patients during COVID-19. Furthermore, there is no published data related to dental patients’ level of HRQoL in Jordan even prior to COVID-19. At the time of data collection, the spread of the COVID-19 was still a risk, according to the reports by health regulatory bodies, and was still far from community outbreak. The government in Jordan took drastic measures to slow the spread of the infection and started preparations to manage this pandemic. These measures mainly focused on closing the borders and limiting gatherings of people by applying curfews, closing schools, universities, and all commercial facilities within the country; this also included health care facilities.^19,30^ During this study, the country was under a total lockdown curfew. The measures taken by the government were bound to have some negative impacts, such as increased stress and depression due to social distancing and isolation, which can affect mental health.^22^ The aim of this study was to evaluate the level of HRQoL and its predictors among dental patients (treatments postponed and dental pain suffered) during COVID-19. Also, this study provides insight into the status of anxiety, stress and depression under the exceptional conditions of the COVID-19 pandemic.^37^

The study results revealed that most patients (43.6%) were largely aware of the seriousness of the COVID-19 pandemic and reported their concerns, although this study was conducted at an early stage of the pandemic in Jordan (from March to May 2020). However, most participants reported only mild anxiety and depression concerning the situation. This may be because from early on, the Jordanian government followed the WHO guidelines and recommendations related to COVID-19.^2^ A high-level collaborative multidisciplinary team, i.e. the National Center for Security and Crisis Management (NCSCM), managed COVID-19 in Jordan using evidence-based recommendations, making sure that the official reports were accessible to the public and effective communication regarding each step was communicated to the general population daily.^2,3^ Several studies and examples of other countries have shown how miscommunication and misinformation due to limited resources or negligence on the part of the regulating bodies caused chaos and stress among the general population.^32,34^

In terms of seeking medical and dental help, very few participants were willing to attend a dental clinic or practice for treatment. Only a few reported that they visited a dental clinic/practice, but only in case of emergency. Less than one-third showed a willingness to attend their appointments to actively undergo dental treatment, if not cancelled due to curfew; the majority of respondents, however, opted to cancel their appointments. These results indicate that patients were seriously concerned regarding infection control in dental clinics. Similar to the results of this study, a cross-sectional study conducted in Saudi Arabia demonstrated that most people avoided seeking dental care due to fear of acquiring COVID-19.^14^ A survey in sub-Saharan African countries studied barriers to seeking medical care in COVID-19, among which included travel restrictions, lockdown and lack of financial means. However, according to the World Bank, Jordan is among the upper-middle-income countries, with a Gross National Income (GNI) of 9430 dollars per capita in 2018.^36^ Therefore, the primary cause of avoiding dental treatment might be fear of contacting COVID-19 or travel restrictions.

Around 84.6% of participants agreed or strongly agreed that dental visits improve their health. Previous studies suggest that patients with dental problems can experience intense dental pain if left to progress with no control or treatment.^8,11^ Here, as the majority avoided seeking dental help, this could be the primary reason for the low level of HRQoL. A mild degree of anxiety was present among all participants of this study regardless of their dental complaints. The study participants reported a greater level of hesitation related to dental visits during the pandemic, as a significant portion of them planned to limit their visits to dental emergencies. The results of this study showed that women are more anxious and have more fear regarding visiting or completing dental treatment based on fear of becoming infected. However, fear of dental treatment is highly prevalent in dental patients regardless of COVID-19.^10^ However, the incidence of COVID-19 might have led to an increase in fear of seeking dental help. Dental associations and dentists should work on this until the public and healthcare professionals become confident in offering treatment under such conditions and the government lifts the curfew to allow patients to visit dental clinics and receive the required treatment.^ 4^

Regardless of sex or region, the feelings about quarantine and the COVID-19 pandemic were significantly associated with the willingness to go to a dental appointment. As the pandemic was still on the rise and out of control worldwide and in Jordan in particular at the time this paper was written, dental healthcare personnel were asked to contact active patients only, treat new patients under utmost adherence to cross-infection control measures, and limit their treatment to relieve the pain of the patient in emergency cases only.^5,26^ In contrast, other countries have taken steps to manage dental outpatient departments during lockdowns. For instance, in Japan, dental telemedicine was introduced to provide online consultancy.^21^ Elsewhere, e-Health and m-health through communication technologies and mobile devices have been highly encouraged for use in daily dental practice in order to manage this pandemic or any other future emergencies.^24^

The data from this study were obtained only from the two largest metropolitan areas in Jordan, leading to unbalanced representation in favour of cities. Nevertheless, our results can be helpful when many dental clinics/practices re-open after lengthy closure, in terms of dentists’ awareness of what to expect in terms of patients’ concerns, e.g. arising from stress, anxiety, and depression. This study is limited by its sample size being moderate and covering only a small portion of the country’s population. The study is also limited by using an online survey, which could have imposed some bias on participants. Finally, the study design does not allow claiming that the mental health symptoms and decline in quality of life are related to the COVID-19 pandemic, as this was a cross-sectional design, and we have no similar data from the pre-pandemic era. Future studies are suggested to target dental treatment alternatives during similar societal emergencies.

## Conclusions

This study highlights how fear related to dental treatment has been enhanced in COVID era and has decreased seeking dental treatment. Additionally, people are experiencing mild depression and anxiety in Jordan. However, compared to other parts of the world, people have less anxiety and stress, probably due to timely and effective management by the government. However, fear of contacting COVID-19 has greatly influenced people’s decision to seek dental treatement, which has negatively impacted the OHRQoL. Implementation of COVID guidelines in dental clinics, along with innovations such as e-health and m-health, might help dentists in encouraging their patients to receive oral treatment.

## References

[ref1] Al Shamrany M (2006). Oral health-related quality of life: a broader perspective. EMHJ.

[ref2] Al-Tammemi AAB (2020). The battle against COVID-19 in Jordan: an early overview of the Jordanian experience. Public Health Front.

[ref3] Alzghol T (2020). Jordan’s Response in Mitigating the COVID-19 Pandemic: The Impact of Public Policy in Addressing a Pandemic. Jerash Res Studies J.

[ref4] American Dental Association (2020). Coronavirus frequently asked questions.

[ref5] Ather A, Patel B, Ruparel NB, Diogenes A, Hargreaves KM (2020). Coronavirus disease 19 (COVID-19): implications for clinical dental care. J Endod.

[ref6] Baiju RM, Peter ELBE, Varghese NO, Sivaram R (2017). Oral health and quality of life: current concepts. JCDR.

[ref7] Broder HL (2001). Using psychological assessment and therapeutic strategies to enhance well-being. Cleft Palate Craniofac J.

[ref8] Campi LB, Visscher CM, Ongaro PCJ, Braido GVV, Fernandes G, Gonçalves DAG (2020). Widespread pain and central sensitization in adolescents with signs of painful temporomandibular disorders. J Oral Facial Pain Headache.

[ref9] Cunha-Cruz J, Hujoel PP, Kressin NR (2007). Oral health related quality of life of periodontal patients. J Periodont Res.

[ref10] Demetriou N, Tsami-Pandi A, Parashis A (1995). Compliance with supportive periodontal treatment in private periodontal practice. A 14-year retrospective study. J Periodontol.

[ref11] Faruqui S, Mubassar F, Attiya S (2018). Factors affecting treatment duration–A dilemma in orthodontics. JAMC.

[ref12] Harari YN (2020). The world after coronavirus. Financial Times.

[ref13] Hescot P (2017). The new definition of oral health and relationship between oral health and quality of life. Chin J Dent Res.

[ref14] Ibrahim MS, Alibrahim H, Al Madani A, Alamri A, Bamashmous M, Tounsi A (2021). Fear factor in seeking dental care among Saudis during COVID-19 pandemic. Int J Environ Res.

[ref15] Jansson H, Wahlin Å, Johansson V, Åkerman S, Lundegren N, Isberg PE, Norderyd O (2014). Impact of periodontal disease experience on oral health–related quality of life. J Periodontol.

[ref16] Jipa I, Amariei C (2012). Oral health-related quality of life: A broader perspective. Acta Medica Transilvanica.

[ref17] Le MTH, Tran TD, Holton S, Nguyen HT, Wolfe R, Fisher J (2017). Reliability, convergent validity and factor structure of the DASS-21 in a sample of Vietnamese adolescents. PloS one.

[ref18] Locker D (1988). Measuring oral health: a conceptual framework. Commun Dent Health.

[ref19] Malkawi SH, Almhdawi K, Jaber AF, Alqatarneh NS (2021). COVID-19 Quarantine-related mental health symptoms and their correlates among mothers: a cross sectional study. Matern Child Health J.

[ref20] Mijiritsky E, Lerman Y, Mijiritsky O, Shely A, Meyerson J, Shacham M (2020). Development and validation of a questionnaire evaluating the impact of prosthetic dental treatments on patients’ oral health quality of life: a prospective pilot study. Int J Environ Res.

[ref21] Morishita M, Takahashi O, Yoshii S, Hayashi M, Kibune R, Nakamura T, Muraoka K, Tominaga K, Awano S (2021). Effect of COVID-19 on dental telemedicine in Japan. J Dent Sci.

[ref22] Msherghi A, Alsuyihili A, Alsoufi A, Ashini A, Alkshik Z, Alshareea E (2021). Mental health consequences of lockdown during the COVID-19 pandemic: a cross-sectional study. Front Psychol.

[ref23] Nettleton S (1993). The sociology of health and illness.

[ref24] Neville P, van der Zande MM (2020). Dentistry, e-health and digitalisation: A critical narrative review of the dental literature on digital technologies with insights from health and technology studies. Commun Dent Health.

[ref25] Parkitny L, McAuley J (2010). The depression anxiety stress scale (DASS). J Physiother.

[ref26] Peloso RM, Pini NIP, Sundfeld Neto D, Mori AA, Oliveira RCGD, Valarelli FP (2020). How does the quarantine resulting from COVID-19 impact dental appointments and patient anxiety levels?. Braz Oral Res.

[ref27] Peng X, Xu X, Li Y, Cheng L, Zhou X, Ren B (2020). Transmission routes of 2019-nCoV and controls in dental practice. Int J Oral Sci.

[ref28] Faul F, Erdfelder E, Buchner A, Lang AG (2009). Statistical power analyses using G* power 3.1: Tests for correlation and regression analyses. Behav Res Methods.

[ref29] Petersen PE, Bourgeois D, Hiroshi O, Estupinan-Day S, Charlotte N (2005). The global burden of oral diseases and risks to oral health. Bulletin of the World Health Organization.

[ref30] Rozier RG, Bhavna TP (2008). Patient- and population-reported outcomes in public health dentistry: oral health-related quality of life. Dent Clin N Am.

[ref31] Saleh OA, Jammal H, Alqudah N, Alqudah A, Abu-Yaghi N (2020). Clinical experience in the administration of intravitreal injection therapy at a tertiary university hospital in Jordan during the COVID-19 lockdown. Clin Ophthalmol.

[ref32] Samani S, Joukar B (2007). A study on the reliability and validity of the short form of the depression anxiety stress scale (DASS-21). Pertanika J Soc Sci Humanit.

[ref33] Setiawana A, Nurmandi A, Purnomo EP, Muhammad A (2021). Disinformation and miscommunication in government communication in handling COVID-19 pandemic. Webology.

[ref34] Shammi M, Bodrud-Doza M, Islam ARMT, Rahman MM (2020). COVID-19 pandemic, socioeconomic crisis and human stress in resource-limited settings: a case from Bangladesh. Heliyon.

[ref35] Sisson KL (2007). Theoretical explanations for social inequalities in oral health. Commun Dent Oral Epidemiol.

[ref36] The World Bank Jordan Profile 2019. https://data.worldbank.org/country/jordan.

[ref37] Vahia IV, Blazer DG, Smith GS, Karp JF, Steffens DC, Forester BP (2020). COVID-19, mental health and aging: A need for new knowledge to bridge science and service. Am J Geriatr Psychiatry.

[ref38] Varul MZ (2010). Talcott Parsons, the sick role and chronic illness. Body Soc.

[ref39] World Health Organization (2020). Coronavirus disease 2019 (COVID-19): situation report.

[ref40] Yazdi FV, Seyfaddini R, Ghandi M, Mehrolhasani MH (2018). The world health organisation’s definition of health: a short review of critiques and necessity of a shifting paradigm. Iran J Epidemiol.

[ref41] Yewe-Dyer M (1993). The definition of oral health. Br Dent J.

